# First report of a short in‐frame biallelic deletion removing part of the EGF‐like domain calcium‐binding motif in LTBP4 and causing autosomal recessive *cutis laxa* type 1C

**DOI:** 10.1002/ajmg.a.62954

**Published:** 2022-08-16

**Authors:** Jean‐Marie Ravel, Margot Comel, Marion Wandzel, Myriam Bronner, Aurélie Tatopoulos, Mathilde Renaud, Laëtitia Lambert, Anne‐Claire Bursztejn, Céline Bonnet

**Affiliations:** ^1^ Laboratoire de génétique médicale CHRU Nancy Nancy France; ^2^ Université de Lorraine, INSERM UMR_S1256, NGERE Nancy France; ^3^ Service de Médecine Infantile CHRU de Nancy Nancy France; ^4^ Service de génétique médicale CHRU de Nancy Nancy France; ^5^ Département de dermatologie CHRU de Nancy Nancy France

**Keywords:** autosomal recessive cutis laxa type 1C (ARCL1C), calcium‐binding epidermal growth factor‐like domain, *LTBP4*, Urban‐Rifkin‐Davis syndrome (URDS)

## Abstract

*Cutis laxa* (CL) is a rare connective tissue disorder characterized by wrinkled, abundant and sagging skin, sometimes associated with systemic impairment. Biallelic alterations in latent transforming growth factor beta‐binding protein 4 gene (*LTBP4*) cause autosomal recessive type 1C *cutis laxa* (ARCL1C, MIM #613177). The present report describes the case of a 17‐months‐old girl with *cutis laxa* together with a literature review of previous ARCL1C cases. Based on proband main clinical signs (cutis laxa and pulmonary emphysema), clinical exome sequencing (CES) was performed and showed a new nine base‐pairs homozygous in‐frame deletion in *LTBP4 gene*. RT‐PCR and cDNA Sanger sequencing were performed in order to clarify its impact on RNA. This report demonstrates that a genetic alteration in the EGF‐like 14 domain calcium‐binding motif of *LTBP4 gene* is likely responsible for cutis laxa in our patient.

## 1 INTRODUCTION

*Cutis laxa* is a group of rare connective tissue disorders characterized by loose and wrinkled skin. It is associated with skeletal and developmental abnormalities and, in some cases, severe systemic impairments (Beyens et al., 2021).

Connective tissue is a supporting tissue consisting of three components: (1) secretory cells synthetizing ground substance and specific proteins which give connective tissue its characteristics, (2) elastin made elastic fibers, responsible for the skin elastic properties, and collagen made reticular fibers contributing to small blood vessels and soft tissues support, and (3) ground substance.

*Cutis laxa* is the Latin term for sagging or slackened, nonstretchable skin. This wrinkle‐like skin can be particularly noticeable on the face, neck, armpits, and limbs. *Cutis laxa* may be inherited or acquired. Acquired forms most often occur in adulthood, their etiology including infections, drugs, or paraneoplasia (Lewis et al., 2004).

Three major groups are individualized based on the inheritance mode: autosomal dominant, autosomal recessive, and X‐linked recessive *cutis laxa*.

Autosomal Dominant Cutis Laxa (ADCL, MIM#123700) is due to pathogenic variations in Elastin (*ELN*, MIM* 130160). ADCL may begin at birth and until early adulthood with predominantly skin symptoms. It is generally a mild form with characteristic facial features and mild to severe systemic manifestations (B. Callewaert et al., 2011). Some rare reports have associated ADCL with *FBLN5* (MIM* 604580, #614434) (Markova et al., 2003) and *ALDH18A1* (MIM* 616603, # 616603) (Fischer‐Zirnsak et al., 2015) pathogenic variants.

Autosomal recessive *cutis laxa* (ARCL) is a very heterogeneous group of diseases, for which three subtypes can be distinguished. ARCL1 manifestations are severe and begin at birth showing aging features and loose skin. Severe systemic complications are frequent and include emphysema and diaphragmatic defects, arterial tortuosity, and aneurysms (de Schepper et al., 2003). Prognostic is severe with death in early childhood due to cardiac or pulmonary complications. ARCL1A is related to pathogenic variants in *FBLN5* (MIM*604580, # 614434) (Markova et al., 2003); ARCL1B to *EFEMP2* (MIM*604633, # 614437) (Hoyer et al., 2009). ARCL1C, also known as Urban‐Rifkin‐Davis syndrome, is caused by biallelic variants in *LTBP4* (MIM*604710, # 613177) (Urban et al., 2009) and is associated with severe pulmonary, gastrointestinal, and urinary abnormalities. There are three forms of ARCL2. ARCL2A includes frequent motor nervous system and cardiovascular abnormalities (Morava et al., 2009) and is caused by pathogenic variations in *ATP6V0A2* (MIM* 611716, # 219200). ARCL2B is associated with *PYCR1*, with main features including abnormal growth, developmental delay and associated skeletal abnormalities (MIM* 179035, # 612940) (Guernsey et al., 2009). ARCL2C, ARCL2D, and ARCL2E are caused by pathogenic variations respectively in *ATP6V1E1* (MIM* 108746, # 617402) (Van Damme et al., 2017), *ATP6V1A* (MIM* 607027, # 617403) (Van Damme et al., 2017) and LTBP1 (MIM*150390, # 619451) (Pottie et al., 2021). ARCL3 is caused by biallelic pathogenic variations in *ALDH18A1* (ARCL3A, MIM*138250, #219150) (Fischer et al., 2014) or PYCR1 (ARCL3B, MIM* 179035, # 614438) (Lin et al., 2011). It corresponds to De Barsy syndrome and includes progeroid features, eye abnormalities, growth retardation, and intellectual disability (Lin et al., 2011).

X‐linked *cutis laxa*, also called occipital horn syndrome (because of exostoses on the occipital bones), involves *ATP7A* (Tang et al., 2006). It belongs to a wide clinical spectrum, including extremely severe Menkes disease.

LTBP4‐related *cutis laxa* is a severe but variable disorder characterized in nearly all patients by *cutis laxa*, severe and progressive pulmonary emphysema, and gastrointestinal complications. Other respiratory features include atelectasis, tracheomalacia and diaphragmatic hernia (Ritelli et al., 2019). Gastrointestinal hernias are common, and pyloric stenosis may occur. Genitourinary abnormalities include diverticulosis and hydronephrosis (Urban et al., 2009). Some cardiovascular defects are also associated with ARCL1C, such as valvular dysfunction and arterial tortuosity (Ritelli et al., 2019). Finally, hypotonia may be evident since birth and be followed by motor development delay.

Here, we report the case of a 17‐month‐old girl born to Algerian‐related parents with autosomal recessive type 1C *cutis laxa* (ARCL1C or Urban‐Rifkin‐Davis syndrome, MIM #613177) due to a biallelic nine base‐pairs in‐frame deletion in *LTBP4*.

## 2 MATERIAL AND METHODS

### 2.1 Editorial policies and ethical considerations

This study was performed as part of routine diagnostic activity. Patient's parents gave their informed written consent and authorized the publication of clinical data and photographs. The patient was clinically evaluated in the dermatology department of Nancy university hospital (France). Genetic analyses were performed at the medical genetics laboratory of Nancy university hospital.

### 2.2 Next‐generation sequencing (NGS)

Molecular analysis was performed on genomic DNA samples purified from peripheral blood leukocytes (Macherey Nagel NucleoSpin® Blood L Vacuum Kit). NGS of a large panel of 4490 genes (Sophia Clinical Exome Solution™, Sophia Genetics SA, Saint Sulpice, Switzerland) was performed (Marinakis et al., 2021). Analysis was realized using manufacturer's bioinformatics products and processing methods (SOPHiA™ DDM version 5.8.0.3, Sophia Genetics SA patented algorithm). In brief, libraries were prepared using random DNA fragmentation. Following the capture of regions of interest and amplification, sequencing was performed on Nextseq 550 Instrument (Illumina, San Diego, CA). Reads were aligned on the reference genome (GRCh37/hg19). Both alignment and variant calling were performed using SOPHiA DDM® platform. Variants were prioritized using SOPHiA DDM® platform based on their consequences on transcripts, interspecies nucleotide conservation, amino acid conservation, in silico functional prediction algorithms and known association with human diseases. Prioritized variants were further analyzed using literature informations and a potential association with the patient's phenotype was assessed. Variations were classified as recommended by the ACMG (American College of Medical Genetics) (Richards et al., 2015). Protein structure prediction was generated with AlphaFold (Jumper et al., 2021) based on uniport number Q8N2S1 and visually assessed.

### 2.3 Sanger DNA sequencing

Conventional PCR and Sanger sequencing were conducted to confirm retained variants and their segregation within the family (genomic primers available upon request). Sequence products were analyzed by capillary electrophoresis on ABI 3130XL (Thermofisher). Results were visualized on the Sequence Scanner software (Thermofisher).

### 2.4 RT‐PCR and sanger sequencing on RNA

According to the usual protocols, RNA extraction from peripheral venous blood was performed with TRIzol. Complementary DNA (cDNA) was obtained using the SuperScript reverse transcriptase reaction (Invitrogen™ Transcriptase inverse SuperScript™ II). We performed reverse transcriptase‐PCR (RT‐PCR) by using the cDNA templates to amplify desired regions of *LTBP4* for the determination of possible splicing changes (primer available on request).

## 3 RESULTS

### 3.1 Clinical description

**FIGURE 1:**
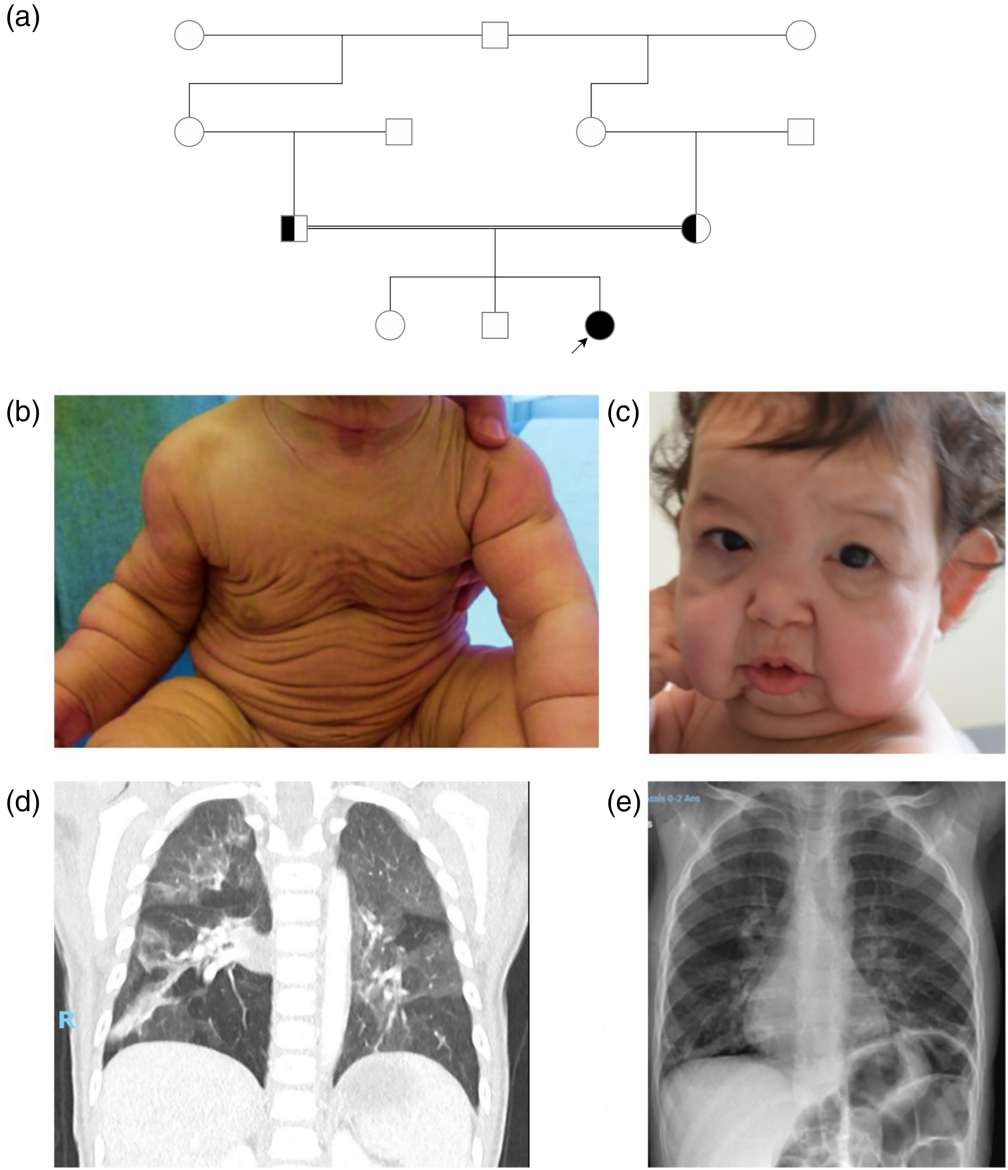
(a) Pedigree. Clinical features (b) loose and flabby skin (c) aged and wrinkled appearance, hypertelorism, periorbital swelling, long philtrum, depressed nasal bridge, microretrognathia, rather large ears, and prominent cheeks due to sagging skin (d) thoracic CT‐scan showing pulmonary emphysema, ventilatory anomalies and a bronchogenic cyst and (e) thoracic X‐ray showing emphysema

We report the case of a third child from an unaffected related couple (their respective mothers are half‐sisters) of Algerian origin (Figure 1a). She was born at a gestational age of 38 weeks with a birth weight of 2820 g (31st percentile), in relation with an intrauterine growth restriction (birth height 47 cm, 24th percentile, head circumference: 33 cm, 30th percentile). On her last clinical examination at 17 months, motor development and physical mensuration were suitable for her age (weight: 9.7 kg, height: 78 cm, mean).

At 6 months, the dermatological examination revealed loose, very elastic, flabby, generalized skin, observed since birth, with an aged and wrinkled appearance (Figure 1b). She presented with periorbital swelling, hypertelorism, long philtrum, depressed nasal bridge, microretrognathia, relatively large ears, and sagging skin with prominent cheeks (Figure 1c). No joint hyperlaxity or hernia, especially umbilical, was noted. At 10 months, thoracic inspection revealed a discreet *pectus excavatum*.

At birth, she had relative pulmonary artery hypoplasia with a normal left ventricular ejection fraction (70%). At 10 months, thoracic CT‐scan and X‐Ray revealed areas of emphysema in both lower lobes and suspicion of mediastinal adenopathies (Figure 1d,e). At the age of 17 months, the mother reported dyspnea on exertion. She noted that the child was polypneic when walking, with increased sweating and slow feeding. CT scan showed a volumetric increase in pulmonary emphysema in the two lower lobes and a liquid lesion of the right costovertebral gutter, suggesting a bronchogenic cyst.

A very slight left lateral‐sternal murmur (1/6) was found at 3 months. Doppler echocardiography revealed minimal hypoplasia in the aortic arch (9 mm in the ascending aorta and 7 mm in the isthmus with an estimated velocity of 2.5 m/s). No cardiac valvulopathy was detected. A slight systolic murmur in the pulmonary area was noted (2/6). Pulmonary arteries appeared hypoplastic on Doppler ultrasound, with the right and left pulmonary arteries measuring between 5 and 6 mm in diameter with a flow velocity accelerated to 2.5 m/s. At 10 months, ultrasonography showed persistent pulmonary arteries hypoplasia with a measure between −2 and −3 *Z*‐scores. Minimal aortic insufficiency over aortic ring dilatation was noted.

### 3.2 Molecular analysis

**FIGURE 2:**
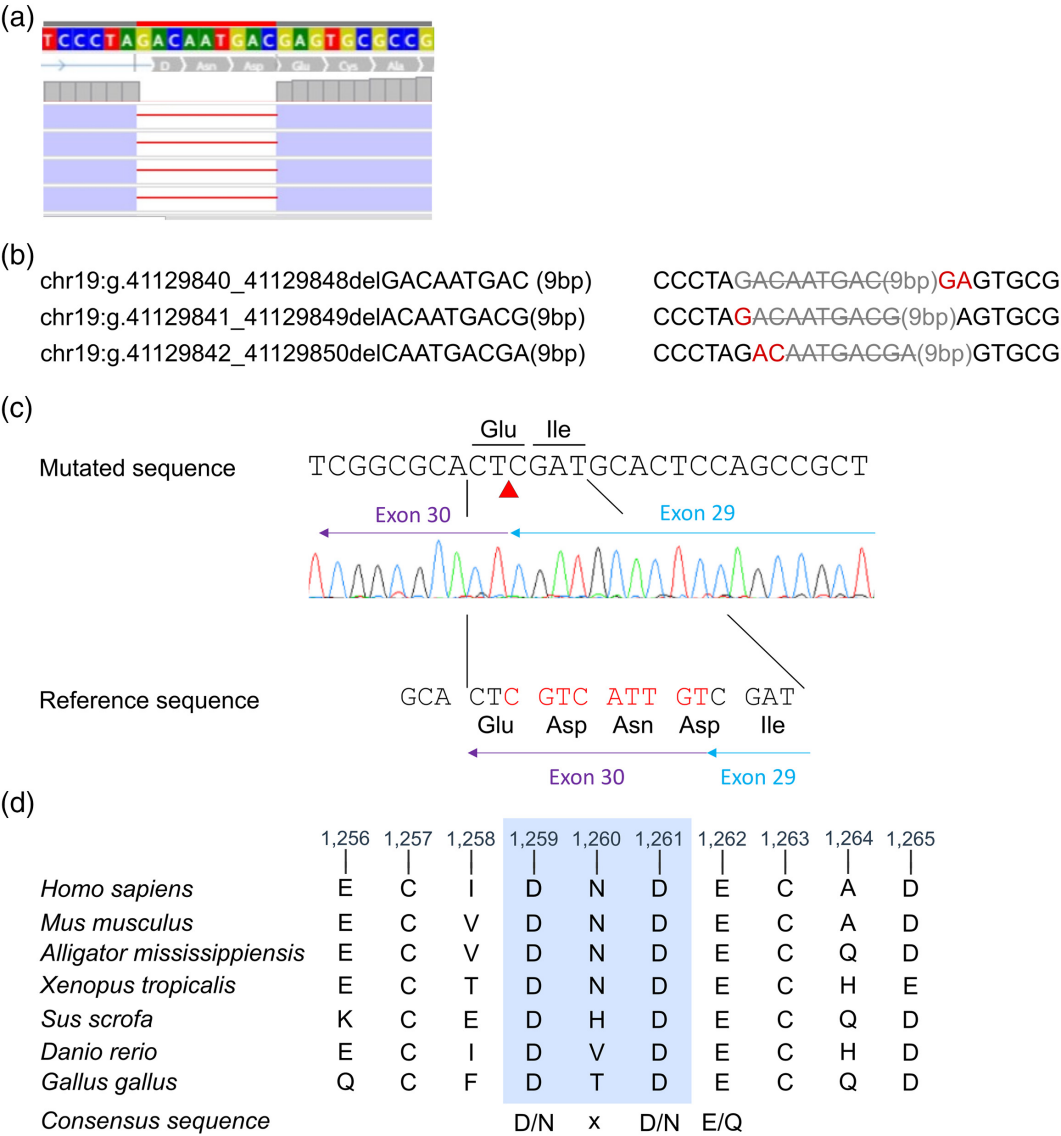
Molecular analysis. (a) Next‐generation sequencing screenshot from IGV showing homozygous deletion of nucleotides “GACAATGAC.” (b) Equivalent deletions (the resulting sequence is the same because of GA motif repetition). (c) Electropherograms from cDNA sequencing showing “CGTCATTGT” nucleotides deletion on reverse strand and leading to the three amino‐acids deletion. The red arrow head indicates the deleted bases localization. (d) BlastP species conservation showing strong conservation of the deleted pattern across vertebrates

Patient DNA sequencing identified a homozygous nine base‐pairs in‐frame deletion at the beginning of *LTBP4* exon 30 (NM_003573.2:c.3774_3782delp.Asp1259_Asp1261del, ClinVar submission SCV002103298). Three deletions are equivalent with the same resulting sequence because of a GA motif repetition in this region (Figure 2a,b). This nine base pairs loss leads a three amino acids loss, resulting in a predicted shorter protein. Segregation of the variant in the family by Sanger sequencing showed that the unaffected parents and the unaffected brother and sister were heterozygous for the *LTBP4* variant allele.

### 3.3 Study of the variant functional impact

In order to confirm our hypothesis that *LTBP4* is the disease‐causing gene in our patient*, w*e performed a RNA analysis of this gene. Because of the proximity of the deletion with the exonic–intronic junction, we hypothesized that exon 30 splicing was affected. A high probability of exon skipping was predicted by in silico prediction tools. Indeed, according to the Ex‐Skip software (https://ex-skip.img.cas.cz/), the variant carrying allele was more likely to being subjected to an exon skipping than the wild type allele. RT‐PCR on RNA was performed with two exonic primer pairs (amplifying exons 28–32 and exons 29–31) and showed no impact on splicing, particularly no exon 30 skipping (data not shown). cDNA Sanger sequencing confirmed the deletion of nine base‐pairs at the beginning of *LTBP4* exon 30 and suggested the loss of three amino acids (p.Asp1259_Asp1261del) (Figure 2c). Protein structure prediction with AlphaFold (Jumper et al., 2021) showed potential hydrogen bond interactions involving residues Asp(D)1259, Asn(N)1260, and Asp(D)1261: between Asp1259 and Gly1278; Asp1257 and Asn1275; Asn 1260 and Glu1262; Asp1261 and Cys1263; Asp1261 and Ala1264.

### 3.4 Analysis of the variation pathogenicity following ACMG guidelines

This variant is absent from the gnomAD database (Pathogenic Moderate 2, PM2). The predicted protein length is shorter than the wild protein and indeed, lacks three amino acids (PM4). The deletion affects the functional EGF‐like 14 domain (PM1). The three deleted amino acids are highly conserved through species (Pathogenic Supporting 3, PP3, Figure 2d). Finally, the patient's phenotype is quite specific to the *LTBP4* gene, for which pathogenic variations are pretty rare (PP4). Our variant, has therefore, two “PP” criteria and three “PM” criteria, which allows its classification as “likely pathogenic,” that is, class 4 according to ACMG classification.

## 4 DISCUSSION

We report here the case of a 17‐months‐old girl with *cutis laxa* for whom a probably pathogenic homozygous variation of *LTBP4*, not reported to date, was identified. The variation is inherited from her two heterozygous related parents.

ARCL1C is a very rare disorder. Only 21 other cases of *LTBP4*‐related *cutis laxa* have been described among 20 families (B. Callewaert et al., 2013; N. Gupta et al., 2020; Ritelli et al., 2019; Su et al., 2015; Urban et al., 2009; Q. Zhang et al., 2020) (Table S1, Figure S1). The case reported here presents the clinical and molecular characteristics of ARCL1C. Our patient physical appearance is in accordance with other reported cases. A typical, generalized *cutis laxa* is present in the patient and is as in all other reported patients (21/21), associated with a prematurely aged appearance. Her craniofacial features are also quite typical of ARCL1C with periorbital swelling (7/11), hypertelorism (12/14), long philtrum (13/16) and depressed nasal bridge (12/14), microretrognathia (9/15) and relatively large ears. This girl's pneumatological presentation includes early pulmonary emphysema with pulmonary arteries hypoplasia, not associated at this time with pulmonary hypertension. Cardiovascular anomalies are less frequent but still present in one‐half of the patient. Hypoplasic aortic arch defect seems not have been yet reported. At the date of this study, patient gastrointestinal and genitourinary assessments did not reveal any anomalies.

From a molecular point of view, sequencing revealed a nine base‐pairs homozygous deletion at the beginning of exon 30 in the index case which is present at the heterozygous state in both parents. This deletion probably leads to the loss of three amino acids and is located in the EGF‐like 14 domain, a highly conserved protein domain in evolution (Downing et al., 1996). No exon skipping was detected; nevertheless, we cannot exclude RNA decay of an aberrant transcript with exon skipping.

LTBP4 is an extracellular matrix protein that is structurally related to fibrillins. LTBP4 regulates fibrilin‐5 dependent elastic fiber assembly (B. L. Callewaert & Urban, 2021; Dabovic et al., 2015). Loss‐of‐function of *LTBP4* has been identified in 20 families. Most of these pathogenic variants result in a premature termination codon and nonsense‐mediated decay: indeed, the mechanism causing cutis laxa is probably the absence of LBTP4 protein, causing failure of fibulin‐5‐elastin complexes to target the microfibrils, resulting in severely impaired elastic fiber formation. Furthermore, only three missense substitutions are reported, which all cause the loss of one of the highly conserved cysteine residues in a TGFβ‐binding (TB) domain or hybrid domain. Loss of these cysteine residues was shown to interfere with LTBP4 conformation and function. Nevertheless, short in‐frame deletion in *LTBP4* has never been described.

Several arguments indicate that this short in‐frame deletion in *LTBP4* is responsible for the cutis laxa in our patient, as stated earlier. More specifically, at the protein level, the Asp1259‐Asn1260‐Asp1261 sequence is located in the calcium‐binding domain of “EGF like 14” domain and their deletion probably drastically alters calcium‐binding. These three residues are highly conserved across species and in other EGF‐like domains containing proteins. Calcium‐binding EGF‐like domains exist as tandem repeats, and calcium‐binding is critical for structural integrity and function (Fehon et al., 1990; Kielty & Shuttleworth, 1993). Calcium binding to the EGF domain plays a role in stabilizing the N‐terminus side of the domain (Selander‐Sunnerhagen et al., 1992). Calcium plays a general role in maintaining the structural rigidity of the protein which is crucial for protein–protein interactions. Protein structure prediction showed potential hydrogen bond interactions involving residues Asp(D)1259, Asn(N)1260, and Asp(D)1261. The deletion probably leads to both altered secondary structure and three‐dimensional folding of amino acids chain.

Moreover, the variation is believed to have significant structural and functional consequences. This hypothesis is strengthened by noticing missense pathogenic variations in the calcium‐binding domain of the EGF‐like domain of *FBN1* in patients with Marfan syndrome (McGettrick et al., 2000; M. Zhang et al., 2021) or *FBN2* in patients with congenital arachnodactyly (P. A. Gupta et al., 2002). The calcium‐binding domain of the EGF‐like domain contains the consensus sequence D/N‐x‐D/N‐E/Q‐Xm‐D/N*‐Xn‐Y/F (where m and n are variable and * indicates possible β‐ hydroxylation). These residues are involved either directly in calcium‐ligation or in calcium‐binding site stabilization (Downing et al., 1996; Handford et al., 1991; Knott et al., 1996; McGettrick et al., 2000; Rao et al., 1995). In a pentagonal arrangement, five side‐chain oxygen ligands stand close to a common plane that includes the central Calcium (Rao et al., 1995). Asp1259‐Asn1260‐Asp1261 deleted in our patient are the three first amino acid of this consensus sequence. Numbers of published calcium binding *FBN1* variations occur in this consensus sequence, particularly in the first D residue or the third D/N residue (McGettrick et al., 2000).

In conclusion, we reported the first case of a biallelic short in‐frame deletion of part of the calcium‐binding motif of an EGF‐like domain in *LTBP4*, causing Autosomal Recessive Cutis Laxa Type 1C. We have shown that variant NM_003573.2:c.3774_3782del p.(Asp1259_Asp1261del) is predicted, with a high degree of confidence, to alter the calcium‐binding motif in the LTBP4 EGF‐like 14 domain. However, further functional studies are required to elucidate the exact pathophysiological mechanism implying this variant.

## CONFLICT OF INTEREST

The authors declare no conflict of interest.

## Supporting information


**Supplementary Table S1** Clinical features of patients with *LTBP4* biallelic pathogenic variations

**Figure S1** Schematic representation of *LTBP4* pathogenic variations associated with ARCL1A based on protein position. The gene consists of four TGF‐beta binding (TB) domains and 16 EGF‐like (E) domains (UniProtKB—Q8N2S1). The variation in bold is the novel variation herein reported. Refseq transcript NM_003573.2 was used as in previous publication except for the NM_001042545.1:c.145_163del p.S(er49Alafs*34) variation that is deeply intronic except in the longest transcript. Mutalyzer was used to convert nomenclature and protein prediction (https://mutalyzer.nl/).

## Data Availability

The data that support the findings of this study are available in ClinVar, reference number SCV002103298.
