# Supplementary material for: First report of a short in‐frame biallelic deletion removing part of the EGF‐like domain calcium‐binding motif in LTBP4 and causing autosomal recessive cutis laxa type 1C
Source: Am J Med Genet A. 2022 Aug 16;188(11):3343–9. doi: 10.1002/ajmg.a.62954 (PMC9805176; doi:10.1002/ajmg.a.62954)
Supplement: Supplementary file 1 — Supplementary Table S1 Clinical features of patients with LTBP4 biallelic pathogenic variations [file AJMG-188-3343-s002.docx]

**Supplementary Table 1: Clinical features of patients with *LTBP4* biallelic pathogenic variations**

Abbreviations: BD, breathing difficulties; BA, brain abscesses; GP, gastric perforation; M, months; NA, not available; PE, pulmonary emphysema; PHrT, pulmonary hypertension; W, weeks; Y, years

| Patients | | P1 | P2 | P3 | P4 | P5 | P6 | P7 | P8 | P9 | P10 | P11 | P12 | P13 | P14 | P15 | P16 | P17 | P18 | P19 | P20 | P21 | P22 |
| --- | --- | --- | --- | --- | --- | --- | --- | --- | --- | --- | --- | --- | --- | --- | --- | --- | --- | --- | --- | --- | --- | --- | --- |
| Citations | | **This report** | Urban et al., 2009 | | | | Callewaert et al., 2013 | | | | | | | | | Su et al., 2015 | | | | Ritelli et al., 2019 | Gupta et al., 2020 | | Zhang et al., 2020 |
| Gender | | F | M | M | F | F | F | M | F | M | M | M | F | F | M | M | F | F | M | F | F | M | F |
| Age | | 17 M | 9 M | 4 M | 7 Y | 26 M | 23 Y | 4 W | 3 M | 2 Y | 10 Y | 6 M | 6 M | 13 Y | 6 W | 15 M | 14 Y | 20 Y | 6W | 18 M | 8 Y | 4 Y | 1 M |
| Cause of death | | - | PE | PE | - | PE | - | PE | PE | PE | PE | PE PHrT | PHrTGP | BA | PE | NA | - | - | BD | - | - | - | - |
| Country | | ALG | ESP | MEX | PLE | ESP | NA | NA | NA | NA | NA | NA | NA | NA | NA | NA | NA | NA | TUR | VN | IN | IN | CN |
| Cutis Laxa | 22/22 | + | + | + | + | + | + | + | + | + | + | + | + | + | + | + | + | + | + | + | + | + | + |
| Joint laxity | 12/21 | - | + | + | + | + | + | - | - | - | - | - | - | - | + | NA | + | + | + | + | + | - | + |
| Consanguinity | 12/22 | + | + | - | + | - | - | + | - | + | + | + | + | + | + | - | - | - | + | + | - | - | - |
| Hypotonia | 11/19 | - | + | + | NA | + | - | + | - | + | - | - | + | - | + | + | NA | NA | + | + | + | - | - |
| **Craniofacial** |  | | | | | | | | | | | | | | | | | | | | | | |
| Long philtrum | 13/16 | + | + | + | + | NA | - | NA | NA | + | NA | NA | + | + | - | - | + | + | NA | + | + | + | + |
| Fat midface | 7/14 | - | + | + | + | NA | - | NA | NA | - | NA | NA | - | - | - | - | NA | NA | NA | + | + | + | + |
| Narrow forehead | 12/15 | - | + | + | NA | + | - | NA | NA | + | NA | NA | + | + | + | - | + | NA | NA | + | + | + | + |
| Periorbital swelling | 7/11 | + | + | + | NA | NA | - | NA | NA | + | NA | NA | + | - | - | + | NA | NA | NA | + | NA | NA | - |
| Hypertelorism | 12/14 | + | + | + | NA | NA | - | NA | NA | + | NA | NA | + | + | - | + | + | + | + | + | NA | NA | + |
| Depressed nasal bridge anteverted nares. | 10/12 | + | + | + | NA | NA | - | NA | NA | + | NA | NA | + | - | + | + | + | NA | NA | + | NA | NA | + |
| Retrognathia, micrognathia | 9/15 | + | + | + | + | + | - | NA | NA | - | NA | NA | - | - | - | - | NA | NA | NA | + | + | + | + |
| **Pulmonary** |  | | | | | | | | | | | | | | | | | | | | | | |
| Tachypnea, respiratory distress | 18/20 | + | + | + | + | + | + | + | + | + | + | + | + | + | + | + | + | NA | NA | + | - | - | + |
| Pneumonia | 7/12 | - | + | - | - | + | NA | NA | NA | NA | NA | NA | NA | NA | NA | + | + | NA | + | + | - | - | + |
| Laryngomalacia, tracheomalacia, bronchomalacia | 5/18 | - | - | + | NA | + | - | + | - | - | - | - | - | - | + | - | NA | NA | NA | - | - | - | + |
| Diaphragmatic hernia or eventration | 10/19 | - | - | + | + | + | + | - | - | + | + | - | + | - | + | + | NA | NA | NA | + | - | - | - |
| Emphysema | 17/18 | + | + | + | NA | + | + | + | + | + | + | + | + | + | + | + | NA | NA | NA | - | + | + | + |
| **Gastrointestinal** |  | | | | | | | | | | | | | | | | | | | | | | |
| Diverticula | 5/18 | NA | - | + | + | NA | - | - | - | - | - | - | + | - | - | + | NA | NA | + | - | - | - | - |
| Intestinal dilatation | 7/18 | NA | + | + | NA | - | - | - | - | - | - | + | - | - | - | + | NA | NA | + | + | - | - | + |
| Rectal prolapse | 3/18 | - | - | - | + | + | - | - | - | - | + | - | - | - | - | NA | NA | NA | NA | - | - | - | - |
| **Genitourinary** |  | | | | | | | | | | | | | | | | | | | | | | |
| Bladder diverticula | 13/19 | NA | + | + | + | NA | + | - | + | - | + | - | - | + | + | + | NA | - | + | - | + | + | + |
| Hydronephrosis | 6/16 | NA | + | + | - | NA | - | + | - | - | - | - | - | - | + | NA | NA | NA | NA | + | - | + | - |
| Inguinal hernia | 4/18 | NA | + | + | - | - | - | - | - | - | + | - | - | - | - | + | NA | NA | NA | - | - | - | - |
| **Cardiovascular** |  | | | | | | | | | | | | | | | | | | | | | | |
| Peripheral pulmonary artery stenosis, pulmonary hypoplasia | 12/21 | + | + | - | NA | + | + | + | - | + | - | - | + | + | - | + | + | + | + | - | - | - | - |
| Atrial septal defect or aneurysms | 6/21 | - | - | - | NA | - | - | - | - | - | + | - | + | - | - | - | - | + | + | + | - | + | - |
| Cardiac valve insufficiency | 5/20 | - | - | - | NA | - | - | - | - | NA | + | - | + | - | + | + | + | - | - | - | - | - | - |
| Pulmonary or aortic valve stenosis | 2/21 | - | - | - | NA | + | - | - | - | - | - | - | - | - | - | + | - | - | - | - | - | - | - |
| Pulmonary hypertension | 8/21 | - | + | - | NA | + | - | - | - | - | - | + | + | + | + | + | - | + | - | - | - | - | - |
| Patent foramen ovale | 5/21 | - | + | + | - | NA | - | - | - | - | - | - | - | - | + | + | - | - | - | - | - | - | + |
