# Supplementary figures and images for: First report of a short in‐frame biallelic deletion removing part of the EGF‐like domain calcium‐binding motif in LTBP4 and causing autosomal recessive cutis laxa type 1C
Source: Am J Med Genet A. 2022 Aug 16;188(11):3343–9. doi: 10.1002/ajmg.a.62954 (PMC9805176; doi:10.1002/ajmg.a.62954)

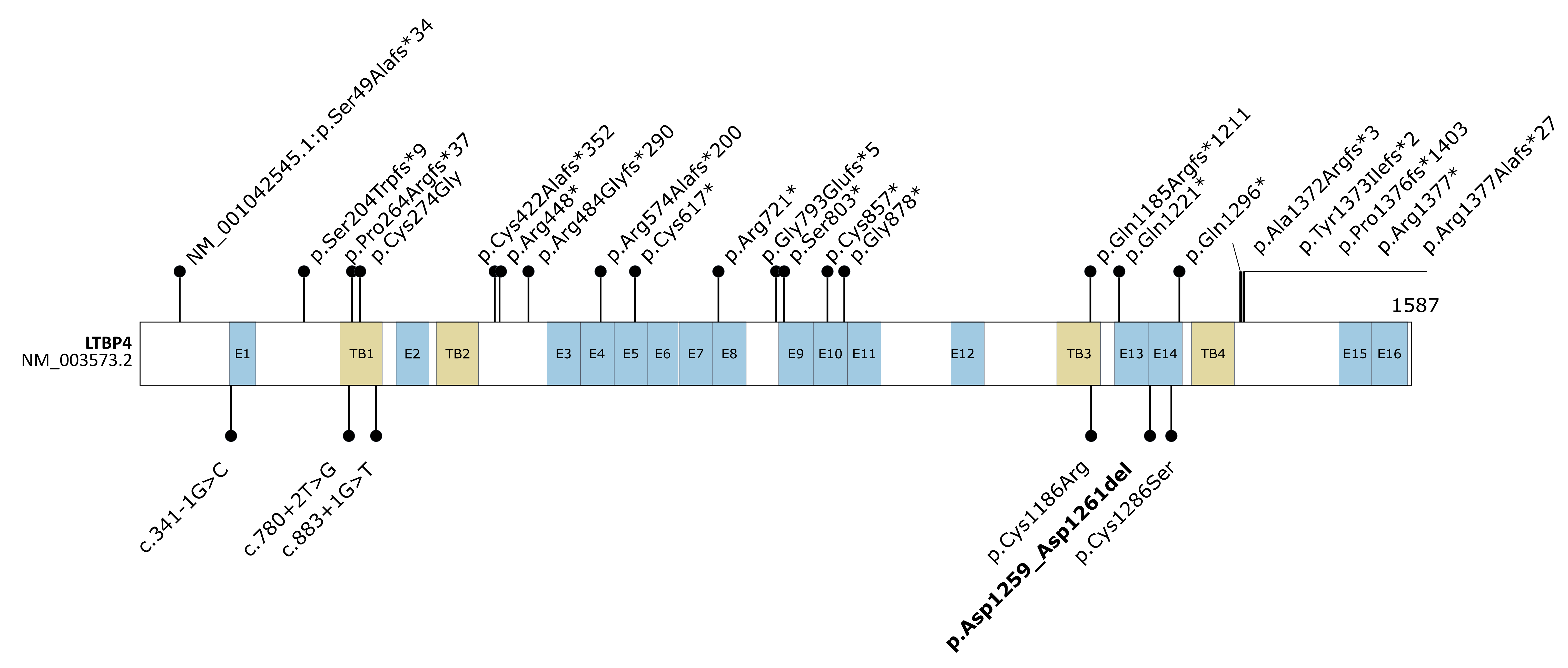

Supplement: Supplementary file 2 — Figure S1 Schematic representation of LTBP4 pathogenic variations associated with ARCL1A based on protein position. The gene consists of four TGF‐beta binding (TB) domains and 16 EGF‐like (E) domains (UniProtKB—Q8N2S1). The variation in bold is the novel variation herein reported. Refseq transcript NM_003573.2 was used as in previous publication except for the NM_001042545.1:c.145_163del p.S(er49Alafs*34) variation that is deeply intronic except in the longest transcript. Mutalyzer was used to convert nomenclature and protein prediction (https://mutalyzer.nl/). [file AJMG-188-3343-s001.png]
